# Significant Decrease in Glycated Hemoglobin, 2h-Post-Load Glucose and High-Sensitivity C-Reactive Protein Levels in Patients with Abnormal Body Mass Index after Therapy with Manual Lymphatic Drainage

**DOI:** 10.3390/biomedicines10071730

**Published:** 2022-07-18

**Authors:** Klaudia Antoniak, Katarzyna Zorena, Marta Jaskulak, Rita Hansdorfer-Korzon, Małgorzata Mrugacz, Marek Koziński

**Affiliations:** 1 Department of Immunobiology and Environment Microbiology, Medical University of Gdansk, Dębinki 7, 80-211 Gdansk, Poland; marta.jaskulak@gumed.edu.pl; 2 Department of Physical Therapy, Medical University of Gdansk, Dębinki 7, 80-211 Gdansk, Poland; rita.hansdorfer-korzon@gumed.edu.pl; 3 Department of Ophthalmology and Eye Rehabilitation, Medical University of Bialystok, Kilinskiego 1, 15-089 Bialystok, Poland; malgorzata.mrugacz@umb.edu.pl; 4 Department of Cardiology and Internal Diseases, Institute of Maritime and Tropical Medicine, Faculty of Health Sciences, Medical University of Gdansk, Powstania Styczniowego 9b, 81-519 Gdynia, Poland; marek.kozinski@gumed.edu.pl

**Keywords:** patients, abnormal body mass index, insulin, 2h-post-load glucose, fasting plasma glucose, C-peptide, high-sensitivity C-reactive protein, lymphatic function, integrative and complementary therapy, manual lymphatic drainage

## Abstract

The objective of this study was to investigate the effect of manual lymphatic drainage (MLD) on the insulin resistance parameter (HOMA-IR), glycated hemoglobin (HbA1c), C-peptide, insulin, fasting plasma glucose (FPG), 2h-post-loadglucose (2h-PG) and the concentration of high-sensitivity C-reactive protein (hsCRP) in patients with abnormal body mass index. The study involved 30 patients, including patients with normal body weight (as a control group; group I; *n* = 14), overweight patients (group II; *n* = 9) and obese patients (group III; *n* = 7). Each patient underwent 10 sessions of MLD therapy, 3 times a week for 30 min. In addition, we measured body mass index (BMI) and waist-to-hip ratio (WHR) and performed body composition analysis as well as biochemical tests before MLD therapy (stage 0′) and after MLD therapy (stage 1′). A statistically significant correlation was demonstrated between the concentration of C-peptide, BMI, the amount of visceral adipose tissue (r = 0.87, *p* = 0.003; r = 0.76, *p* = 0.003, respectively), and the HOMA-IR index, BMI and the amount of visceral adipose tissue (r = 0.86, *p* = 0.005; r = 0.84, *p* = 0.042, respectively), before and after MLD therapy. In overweight patients (group II), a statistically significant (*p* = 0.041) decrease in the hsCRP level by 2.9 mg/L and a significant (*p* = 0.050) decrease in the 2h-PG level by 12 mg/dL after the MLD therapy was detected. Moreover, in the group of obese patients (group III), a statistically significant (*p* = 0.013) decrease in HbA1c level by 0.2% after MLD therapy was demonstrated. Our results indicate that MLD may have a positive effect on selected biochemical parameters, with the most favorable changes in overweight patients. Further studies in a larger number of patients are warranted to confirm our findings, to test in-depth their mechanism, and to investigate clinical benefits of this alternative therapy in patients with abnormal body mass index.

## 1. Introduction

Obesity is a chronic disease with a complex etiology, defined as the abnormal or excessive accumulation of adipose tissue [1], affecting (along with overweight) more than one third of the world’s population [2,3]. It is estimated in recent reports that by 2030 approximately 38% of the global adult population will be overweight, and another 20% will be obese [4]. As the prevalence of obesity increases, more and more studies show that this pathological condition has a detrimental effect on various organ systems, and the complications resulting from these consequences are a frequent cause of morbidity and mortality [5,6,7,8,9]. People with abnormal vs. normal body mass index have a significantly greater risk of developing diabetes [6], hypertension [7], ischemic heart disease [8], and atherosclerosis [9]. To assess the risk of developing obesity-related diseases, not only anthropometric indicators can be used [10], but also biochemical markers, e.g., high-sensitivity C-reactive protein (hsCRP), fasting plasma glucose (FPG) and/or 2h-post-loadglucose (2h-PG), or glycated hemoglobin (HbA1c) [11,12,13]. C-reactive protein is sensitive and the most accessible biomarker used for detection and monitoring of inflammation [14].

Assessment of glucose metabolism is traditionally based on FPG and/or 2h-PG after a 75 g oral glucose tolerance test (OGTT) [11]. Increased levels of FPG and 2h-PG have been shown to be strong risk factors for the development of T2DM and cardiovascular disease (CVD) by promotion of oxidative stress, inflammation and endothelial dysfunction [15]. Additionally, earlier studies have shown that an increased concentration of 2h-PG in the oral glucose tolerance test more strongly predicts the risk of subsequent cardiovascular disease than increased FPG [12,16]. Therefore, it is important to test not only FPG but also the concentration of 2h-PG. Therefore, in Diabetes Epidemiology: Collaborative Analysis of Diagnostic Criteria in Europe (DECODE), data from 10 prospective European cohort studies were analyzed. The authors showed that an increased level of 2h-PG was a stronger predictor than an increased level of FPG in all-cause mortality, as well as CVD. It should be noted that the highest number of excessive deaths was observed in patients with impaired glucose tolerance (IGT) after 2h-PG, but with normal FPG levels [17]. Additionally, in 2010, the American Diabetes Association [18], and in 2013, the European Diabetes Association, recommended the inclusion of HbA1c concentration testing, a biomarker recognized as a retrospective glycemic index, to be used as a marker for the detection of glucose homeostasis disorders [19]. In a prospective study, it was shown that a 1% increase in HbA1c concentration is associated with an approximately 30% increase in all-cause mortality and a 40% increase in mortality due to cardiovascular or ischemic heart diseases among subjects with/without diabetes [20], while reducing HbA1c levels by 0.2% may lower mortality by 10% [20].

In order to prevent the development of obesity as well as its chronic complications, many pharmacological and non-pharmacological strategies are used [21,22,23,24].

However, considering the side effects of pharmacological treatment and the difficulty in maintaining a long-term restrictive diet, non-pharmacological interventions and lifestyle changes are gaining more and more attention [23]. In recent years, health care professionals have started to pay particular attention to the role of the lymphatic system in the course of obesity [25,26,27,28]. Several studies, including ours, indicate that obesity may cause pathological changes in the lymphatic system that may impair its function, and vice versa—dysregulation of the lymphatic system may lead to the pathogenesis of obesity [28,29]. In our preliminary studies (three cases with a 3-month follow-up), an improvement in the C-peptide concentration and lipid profile was observed, as well as reduction of inflammation and improvement in the quality of life of patients with an abnormal body mass index after manual lymphatic drainage (MLD) therapy [28].

MLD therapy is used to improve lymph flow in the lymphatic system and to discharge metabolites into the circulatory system and then into the excretory system [30,31]. MLD therapy can also stimulate the flow of nutrients transported through the blood to the tissues, as well as improve the metabolism of adipose tissue and the drainage of metabolic products [32]. The effect of MLD on the lymphatic system has been shown in several imaging studies [30,33,34]. In a study of de Godoy et al. using lymphoscintigraphy, an improvement was shown in the transport of radiotracers in lymphatic collectors after MLD therapy in patients with lymphedema [30]. Moreover, in other studies, the assessment of the effectiveness of MLD was conducted by using the fluorescence lymphography method [33,34]. The studies, during and after MLD therapy, showed increased contractility of lymphatic vessels in healthy subjects [33] as well as in patients with lymphedema [33,34]. A more detailed description of imaging studies is available in our previous publication [29].

So far, the main application of MLD has been to support the treatment of lymphedema, venous edema or lipedema [35,36]. Only in recent years have there been papers indicating the use of MLD in supporting the therapy for type 1 diabetes (T1D) [37] or T2DM [38]. Based on a few reports, it has been hypothesized that MLD may be a beneficial therapy in preventing the development of obesity and thus improving the quality of life in patients with abnormal body mass index. That is why the aim of our study was to investigate the effect of MLD on the insulin resistance parameter (HOMA-IR), HbA1c, C-peptide, insulin, FPG, 2h-PG and the concentration of hsCRP in patients with abnormal body mass index.

## 2. Materials and Methods

### 2.1. Data Collection

**Figure 1:**
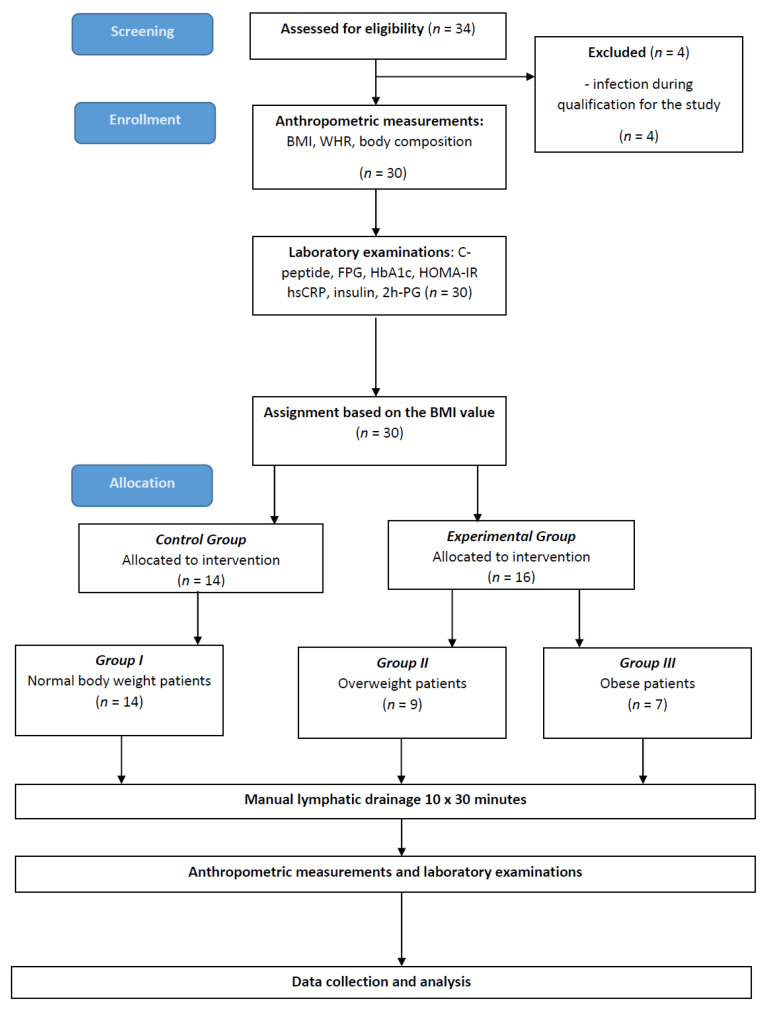
Study flow chart. Abbreviations: BMI, body mass index; FPG, fasting plasma glucose; HbA1c, glycated hemoglobin; HOMA-IR, homeostatic model assessment–insulin resistance; hsCRP, high-sensitivity C-reactive protein; WHR, waist–hip ratio; 2h-PG, 2h-post-load glucose.

The study was approved by the Ethics Committee of the Medical University of Gdańsk (approval no. NKBBN/692/2019–2020; approval date: 30 January 2020), and the investigation was carried out in accordance with the principles of the Declaration of Helsinki as revised in 2013. The subjects were recruited from among patients who came to the Department of Cardiology and Internal Diseases in the Institute of Maritime and Tropical Medicine in Gdynia, through physiotherapy clinic and social media. Thirty-four candidates were screened for this study enrolment. Four of them were excluded due to their inability to participate in all of the steps, for reasons related to infection. As a result, 30 people completed all stages of the research. The patients were examined according to the scheme presented in Figure 1.

The mean age of subjects was 40 ± 12 years, body mass index (BMI) = 27 ± 5 kg/m^2^, the mean of the waist–hip ratio (WHR) was above the limit of abdominal obesity and amounted to 0.92 ± 0.16, and the mean of the visceral adipose tissue level of subjects was 7 ± 4 LVL. The mean blood pressure was 128/88 mmHg. The degree and type of obesity were determined on the basis of the BMI and WHR values according to the guidelines of the Polish Diabetes Society [39]. The patients enrolled in the study according to the guidelines of the Polish Diabetes Society were divided into three groups: patients with normal body weight (as a control group; group I), patients that were overweight (group II), and patients with obesity (group III).

The inclusion criteria were: (1) adults (age >18 years); (2) impaired glucose tolerance; (3) insulin resistance and/or hyperinsulinemia; (4) not taking medications able to modify glucose metabolism (e.g., antipsychotics, cortisone, diuretics, transcriptase or protease inhibitors) in the previous 3 months; (5) no significant changes in dietary regimen in the previous 3 months; (6) BMI from 20 to 35 kg/m^2^. The exclusion criteria were (1) T1D/T2DM; (2) uncontrolled arterial hypertension; (3) clinically relevant cardiac arrhythmia; (4) deep and superficial vein thrombosis;(5) acute kidney injury; (6) acute liver failure; (7) neoplastic diseases; (8) autoimmune diseases; (9) infectious diseases; (10) contraindications to the use of MLD, such as dermatitis or hematomas on the neck and /or abdominal cavity, inability to participate in physiotherapy treatments, and/or inability to understand the instructions. All of the patients received oral and written information about the study, and written informed consent forms were obtained prior to enrolment in the study.

### 2.2. Anthropometric Measurements

Each patient had their anthropometric measurements taken with the usage of the same scale. The anthropometric measurement procedure was described in detail in our previous publication [28]. The Tanita SC-240 foot-to-foot body composition analyzer (Tanita Cooperation, Tokyo, Japan) was used to assess bioelectrical impedance. Measurements were collected at 50 Hz using the standard setting after manually imputing the measured gender, age, and height of the patient. The patients wore minimal clothing, were barefoot, and were commanded to standstill with their feet in direct contact with all four metal plates. The level of the visceral adipose tissue was calculated using the internal equations of the Tanita analyzer. The anthropometric measurements in patients were performed at 2 stages: at stage 0′ (before therapy) and at stage 1′ (one month after therapy).

### 2.3. Laboratory Examinations

After qualification, on the first visit each patient underwent a biochemical test. The concentrations of FPG, HbA1c, hsCRP, and C peptide were measured using methods described in our previous study [28]. The 2h-PG concentrations were measured using the hexokinase method and spectrophotometry (Cobas 8000 analyzer, Roche, Basel, Switzerland). Insulin concentrations were assessed with a chemiluminescent immunometric assay (Cobas e601; Roche, Basel, Switzerland). As an insulin resistance indicator, the homeostasis model assessment for insulin resistance (HOMA-IR) [40] was performed according to the formula: HOMA-IR = (FPG × fasting serum insulin)/405). The units of FPG and fasting serum insulin for the calculation of HOMA-IR were mg/dL and µIU/mL, respectively.

The biochemical parameters in patients was performed in 2 stages: stage 0′ (before therapy) and stage 1′ (one month after therapy).

### 2.4. Interventions

The MLD therapy sessions were conducted in a quiet physiotherapy room with the temperature kept at 22–24°C to reduce the influence of the environment on physiological responses.

During each physiotherapeutic visit, blood pressure was assessed (described in detail in the previous publication [28]). Each subject underwent 10 MLD therapy sessions according to Földi [41], which covered the abdominal cavity, groin region and the neck region. First, the neck region was pre-treated with MLD therapy, and subsequently, the abdominal cavity and groin region were treated with MLD therapy. Then, abdominal lymph nodes were stimulated by deep abdominal drainage. During the abdominal MLD therapy, subjects performed deep breathing.

The complete procedure was as follows:

Pretreatment: Manual lymphatic drainage of the neck; effleurage: superficial strokes from the sternum to the acromion →stationary circles in the supraclavicular fossa on the lower deep cervical lymph nodes → stationary circles from the mandibular angle, over the upper and lower deep cervical lymph nodes → stationary circles along the nuchal line, occipital lymph nodes → stationary circles in front of and behind the ear → stationary circles from the acromion posteriorly to the spine of the scapula to the supraclavicular fossa → stationary circles in the supraclavicular fossa → superficial strokes from the sternum to the acromion.

Manual lymphatic drainage of the abdomen: effleurage: during inhalation, rotary technique from the pubic bone to the sternum → during exhalation, rotary technique from the costal arch and the iliac crest back toward the pubic bone → circles over the cisterna chyli and the course of the large intestine → stationary circles over the descending, ascending and transverse colon, and the pressure is applied in the direction of the cisterna chyli → stroke of seven: circular strokes over the descending colon from the spleen area to the bladder → circular strokes over the ascending colon to the liver area → stationary circles over the navel and return to the spleen area → treatment of the iliac lymph nodes.

Abdominal deep drainage: on the navel → parallel to the left costal arch → parallel to the left inguinal ligament → parallel to the left costal arch → on the navel → parallel to the right costal → parallel to the right inguinal ligament → parallel to the right costal arch → on the navel → conclusion effleurage with breathing [41]. The MLD procedure with photos was presented in detail in our previous publication [28]. MLD therapy was conducted by one experienced physical therapist; one-time therapy lasted 30 min and was carried out three times a week. All study participants were asked to maintain their normal lives and not to engage in any exercise or diet programs that would change their weight [28].

### 2.5. Statistical Methods

In order to determine the normality of data, the Shapiro–Wilk test was used. The results that met the criteria of normal distribution were described as means ± SD. Intergroup and intragroup calculations are presented as means and standard deviations (SD). Correlations between C-peptide, HOMA-IR, hsCRP and BMI, WHR, and the level of visceral tissue were analyzed by Pearson’s correlation. Comparisons between MLD therapy and post-MLD therapy by the *t*-test for dependent samples were performed for parametric results. A significance level of *p* ≤ 0.05 was set for all statistical analyzes. All data were processed with OriginPro 2021 version 9.8 (OriginLab Corporation, Northampton, MA, USA).

## 3. Results

### 3.1. Subjects’ Clinical Characteristics

Thirty subjects were finally included in the study (Figure 1). The mean age of subjects was 40 ± 12 years, BMI = 27 ± 5 kg/m^2^, the mean of the WHR was above the limit of abdominal obesity and amounted to 0.92 ± 0.16, the mean of the visceral adipose tissue level of subjects was 7 ± 4 LVL. In the group of subjects before MLD therapy, mean FPG level was 91 ± 15 mg/dL, 2h-PG level 93 ± 20 mg/dL, mean insulin level 10 ± 8 uIU/mL, mean C-peptide level 2.3 ± 1.9 ng/mL, mean HbA1C level 5.3 ± 0.4%, mean hsCRP level 2.7 ± 5 mg/L, and mean HOMA-IRlevel 2.6 ± 2.1 ng/mL. The characteristics of the subjects are presented in Table 1.

### 3.2. The Correlations between the Level of C-peptide and BMI, WHR and the Level of Visceral Tissue in Patients with Normal Body Weight, Overweight and Obesity before and after MLD Therapy

We found a very strong correlation between the concentration of C-peptide and BMI (r = 0.87, *p* = 0.003) and strong correlation between the concentration of C-peptide and the level of visceral tissue (r = 0.76, *p* = 0.003) in all groups (*n* = 30) before and after MLD therapy. However, no correlation was found between the concentration of C-peptide and WHR (r = −0.018, *p* = 0.920) in all groups (*n* = 30) before and after MLD therapy. The correlations in graphical form are presented in Supplementary Figure S1.

### 3.3. The Correlations between the Level of HOMA-IR and BMI, WHR and the Level of Visceral Tissue in Patients with Normal Body Weight, Overweight and Obesity before and after MLD Therapy

Moreover, the study showed a very strong correlation that was statistically significant between the level of HOMA-IR and BMI (r = 0.86, *p* = 0.005) and between the level of HOMA-IR and visceral tissue level (r = 0.84, *p* = 0.042) in all groups (*n* = 30) before and after MLD therapy.

In contrast, we failed to demonstrate a correlation between the concentration of C-peptide and WHR (r =−0.018, *p* = 0.920) in all groups (*n* = 30) before and after MLD therapy. The correlations in graphical form are presented in Supplementary Figure S2.

### 3.4. The Correlations between the Level of hsCRP and BMI, WHR and the Level of Visceral Tissue in Patients with Normal Body Weight, Overweight and Obesity before and after MLD Therapy

Additionally, the study showed a weak correlation between the concentration of hsCRP and BMI (r = 0.29, *p* = 0.322), very weak correlation between the concentration of hsCRP and WHR (r = 0.11, *p* = 0.930), and moderate correlation between the concentration of hsCRP and the visceral tissue level (r = 0.41, *p* = 0.184) in all groups (*n* = 30) before and after MLD therapy. However, no statistically significant correlations were detected between the concentration of hsCRP and BMI, WHR and the visceral tissue level in all groups.

### 3.5. The Characteristics of Patients with Normal Body Weight as a Control Group (Group I), Patients with Overweight (Group II) and Patients with Obesity (Group III)

In group I, 14 subjects were enrolled (*n* = 14); their mean age was 37 ± 13 years, body mass index (BMI) = 22 ± 2 kg/m^2^, the mean of the WHR (waist–hip Ratio) was below the limit of abdominal obesity and amounted to 0.78 ± 0.075, and the mean of the visceral adipose tissue level was 4 ± 2 LVL. In group II, 9 subjects were enrolled (*n* = 9); their mean age was 40 ± 11 years, BMI = 28 ± 2 kg/m^2^, the mean the WHR was above the limit of abdominal obesity and amounted to 1, and the mean of the visceral adipose tissue level was 8 ± 4 LVL. In group III, 7 subjects were enrolled (*n* = 7); their mean age was 46 ± 10 years, BMI = 34 ± 2 kg/m^2^, the mean of the WHR was above the limit of abdominal obesity and amounted to 1.2 ± 0.1, and the mean of the visceral adipose tissue level was 12 ± 4 LVL. The characteristics of the studied groups are presented in Table 2.

### 3.6. Levels of Biochemical Parameters before (0′) and One Month (1′) after the Use of Manual Lymphatic Drainage Therapy in Patients with Normal Body Weight (Group I)

As a result of the MLD therapy in the group with normal bodyweight, no changes in the concentration of FPG, 2h-PG and hsCRP were observed, where as there were decreases in the concentrations of insulin (8.3 vs. 4.9 uIU/mL after MLD therapy), C-peptide (1.9 vs. 1.3 ng/mL after MLD therapy) and HbA1C (5.2 vs. 5.1% after MLD therapy); however, the differences were not statistically significant (respectively *p* = 0.122, *p* = 0.220 and *p* = 0.613). Moreover, in group I (patients with normal body weight) a decrease in the HOMA-IR level (2 vs. 1.1; *p* = 0.112) after MLD therapy was observed, but this result was not statistically significant. The results for patients with normal bodyweight are shown in Table 3.

### 3.7. Levels of Biochemical Parameters before (0′) and One Month (1′) after the Use of Manual Lymphatic Drainage Therapy in Overweight Patients (Group II)

Following MLD therapy in the subgroup of overweight patients, statistically significant decreases in the concentrations of 2h-PG (112 vs. 100 mg/dL, *p* = 0.050) and hsCRP protein (5.2 vs. 2.3 mg/L; *p* = 0.041) were observed. The statistically significant results are also presented in graphical form in Supplementary Figure S3. Moreover, in the group of overweight patients, we detected decreases, albeit not statistically significant, in the concentrations of insulin (12 vs. 10 uIU/mL) and C-peptide (3 vs. 2 ng/mL), as well as in the level of HOMA-IR after MLD therapy. The results for group II are presented in Table 4.

### 3.8. Levels of Biochemical Parameters in Subgroup III after the Use of Manual Lymphatic Drainage Therapy

Following MLD therapy in the subgroup of obese patients, a statistically significant decrease in the concentration of HbA1C (5.4 vs. 5.2%, *p* = 0.013) was observed. The statistically significant results are also presented in graphical form in Supplementary Figure S4.

The results for group III are presented in Table 5.

### 3.9. Average Differences in the Biochemical Parameters in Groups I, II and III before and after the Use of Manual Lymphatic Drainage Therapy

**Figure 2:**
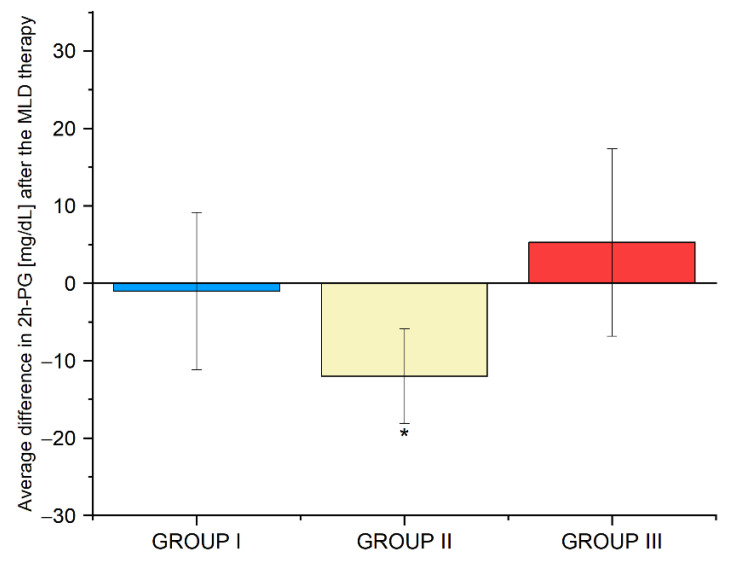
Average differences in 2h-PG levels in group I, II and III before and after the use of MLD therapy. Abbreviations:2h-PG, 2h-post-load glucose, * *p*-value—significant difference (*p* < 0.05).

In groups I, II and III, no statistically significant changes in the concentrations of FPG, insulin, and C-peptide and the level of HOMA-IR were observed. The mean difference in 2h-PG levels, before and after MLD therapy, was −1 mg/dL (*p* = 0.864) in group I,−12 mg/dL (*p* = 0.050) in group II, and + 7 mg/dL (*p* = 0.433) in group III. The results are shown in Figure 2. Detailed data are provided in Supplementary Table S1.

**Figure 3:**
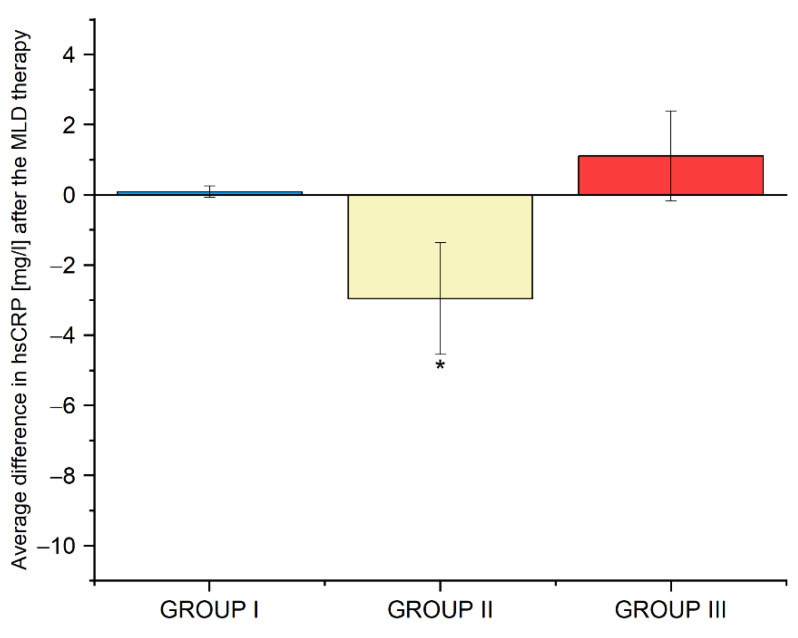
Average differences in hsCRP levels in groups I, II and III before and after the use of MLD therapy. Abbreviations: hsCRP, high-sensitivity C-reactive protein, * *p*-value—significant difference (*p* < 0.05).

The mean difference in hsCRP levels, before and after MLD therapy, was 0 mg/L (*p* = 0.562) in group I−2.9 mg/L (*p* = 0.041) in group II, and + 1.5 mg/L (*p* = 0.480) in group III. The results are shown in Figure 3. Detailed data are provided in Supplementary Table S2.

**Figure 4:**
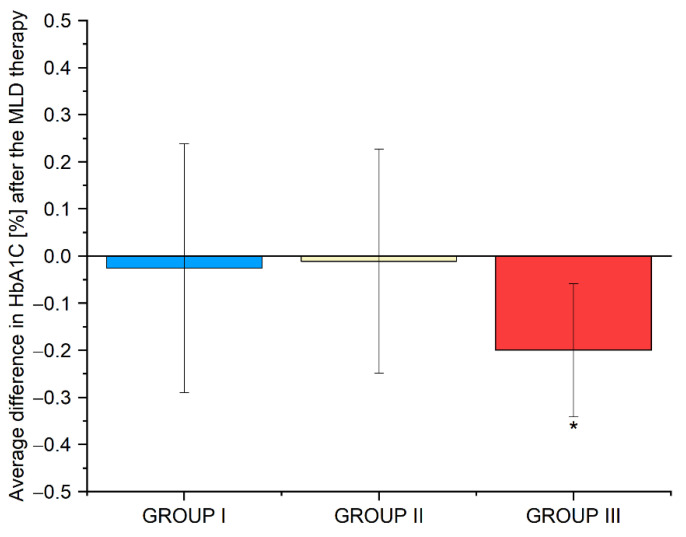
Average differences in HbA1c levels in groups I, II and III before and after the use of MLD therapy. Abbreviations: HbA1c, glycated hemoglobin; * *p*-value—significant difference (*p* < 0.05).

The mean difference in HbA1C levels, before and after MLD therapy, was −0.1% (*p* = 0.613) in group I, 0% (*p* = 0.931) in group II, and−0.2% (*p* = 0.013) in group III. The results are shown in Figure 4. Detailed data are provided in Supplementary Table S3.

## 4. Discussion

In this study, we examined the effect of MLD on biochemical parameters in subjects with abnormal body weight. The examined patients were divided into three groups: patients with normal body weight (group I), overweight patients (group II), and obese patients (group III). One of the parameters we assessed was the level of C-peptide as an indicator of insulin resistance and a marker of pancreatic beta cells function [42]. Our study showed a decrease in the level of C-peptide concentration after MLD therapy in the group of patients with normal body weight and in the group of overweight patients. However, it should be added that the level of C-peptide was within the reference norm, both before and after MLD therapy. A decrease in the level of insulin concentration was observed in group I and group II. Simultaneously, our study showed that before MLD therapy, the value of the HOMA-IR index was above the norm in each of the studied groups. The mean differences between the HOMA-IR index values before and after MLD therapy were greatest in group I, followed by group II and group III, respectively. To the best of our knowledge, we are the first team to assess the impact of MLD therapy on parameters such as C-peptide, insulin and the HOMA-IR index in patients with abnormal body weight. The results of our research may indicate the need for therapies aimed at improving the function of the lymphatic system, as suggested also in the study by Cao et al. [43]. In this study, the authors showed that the lymphatic system has been indicated as the main therapeutic target in the treatment of the consequences of obesity, including insulin resistance [43]. In turn, in experimental research, the authors assessed the effect of abdominal massage on the insulin level and HOMA-IR index in rats fed a high-fat diet. The results of this study showed a decrease in insulin levels and the HOMA-IR index in the abdominal massage group of rats fed a high-fat diet compared to the control group [23].

These findings provide novel insights on the extension of the application of MLD therapy as a potential therapy for improving insulin sensitivity of the tissue in patients with IR, as well as in the prevention of lymphedema in patients with abnormal body weight.

It should be noted that the changes in the concentration of HbA1C after MLD therapy in the studied patients was an important finding of our research. According to the guidelines of the Polish Diabetes Association (PTD), in line with the recommendations of the American Diabetes Association (ADA), the HbA1C parameter is a clinically recognized risk factor for the development of diabetes and/or chronic vascular complications [18,39]. Therefore, we also tried to assess HbA1c levels both before and after MLD therapy in all of our patients. In the study conducted before MLD therapy, the level of HbA1c was within the reference range in all study groups, with the highest value of 5.4% in the obese group and the lowest, of 5.2%, in the subjects with normal body weight. However, it was in the group of obese patients that a statistically significant decrease in HbA1c concentration, by 0.2%, after MLD therapy was observed. Although the level of HbA1c was within the reference range, the decrease in the value seemed to be of clinical significance, which was confirmed by the research of other authors [20,44,45,46]. In a prospective study conducted on 4662 subjects, the lowest mortality rate was demonstrated in subjects with HbA1c levels below 5%. In addition, it has been estimated that in subjects with HbA1c levels between 5–7%, lowering the average HbA1c concentration by 0.1% may prevent an excessive number of deaths by as much as 12%, or as much as 25% if the concentration is reduced by 0.2% [20]. Moreover, studies conducted so far have shown that a decrease in HbA1c levels reduces the risk of developing chronic vascular complications [45,46]. Therefore, it is very important to strive for normal HbA1c values in patients with abnormal body mass index. We suggest that the use of MLD therapy in obese patients may prevent the risk of developing chronic vascular complications and/or T2DM, as well as reducing the risk of premature death. Our results can be compared with the studies by de Sire et al., whose pilot study was conducted in a group of 30 patients with lymphedema and T2DM. Tested T2DM patients were subjected to three sessions of MLD therapy a week for 4 weeks. The study showed a statistically significant decrease in HbA1c and indicated MLD therapy as the recommended therapy for lowering glycemic parameters in patients with lymphedema and T2DM [38].

Finally, among the crucial findings in the group of overweight subjects, a statistically significant decrease in the concentration of 2h-PG by 12 mg/dL and a significant decrease in hsCRP by 2.9 mg/L were observed after the application of MLD therapy. To our knowledge, apart from the previous study by Antoniak et al., which also detected a decrease in hsCRP concentration after MDL therapy in an overweight patient, no team undertook to assess the effect of MLD therapy on hsCRP levels in patients with abnormal body weight. The relationship between the concentration of acute phase proteins, clinical inflammation marker (hsCRP) and the level of 2h-PGmay be explained by the fact that hyperglycemia contributes to increased inflammation and atherosclerosis by increasing the level of oxidative stress and inflammatory markers [47,48]. Brownlee et al. proposed the role of postprandial hyperglycemia in promoting the overproduction of reactive oxygen species [49]. Excess reactive oxygen species indirectly accelerate inflammation in atherosclerosis by increasing ox-LDL formation, promoting insulin resistance, and reducing the activation of adiponectin, AMP-activated protein kinase and endothelial nitric oxide synthase (eNOS) [50]. Additionally, in a prospective epidemiological study, hsCRPwas identified as an independent risk factor for cardiovascular disease (CVD) [13]. Low, moderate and high risk of CVD correlates with hsCRP values <1.0, 1.0 to 3.0 and >3 mg/L, respectively [14,51]. Thus, the decrease in hsCRP we observed in overweight patients may be of clinical significance and contribute to a reduction in the risk of CVD.

Previous studies suggest that obesity-induced lymphatic damage may exacerbate metabolic abnormalities by increasing systemic and local inflammatory responses and regulating insulin sensitivity. These findings suggest that manipulation of the lymphatic system may be an additional therapy to support the treatment of obesity-related metabolic disorders [52,53]. Moreover, the effect of MLD on carbohydrate parameters has also been demonstrated in patients with T1D [37]. The authors showed that MLD therapy for the lower limbs leads to a reduction in blood and urine glucose levels in patients with T1D. It was also suggested that due to the effect of MLD therapy on fluid filtration, excess glucose could be drained through the urinary tract [37]. Research results may indicate the effectiveness of MLD, not only in preventing the development of the consequences of obesity, including insulin resistance and T2DM, but also in supporting the treatment of T1D. This highlights the need for further research on more patients with an abnormal body mass index.It needs to be highlighted that the relationship between disorders of the lymphatic system and abnormal body weight is complex. We can explain the greater improvement of biochemical parameters in overweight patients in several ways. Mak et al. indicated a functional link between lymphatic dysfunction and the pathogenesis of obesity. It has been suggested that patients with higher BMI may need greater blood and lymphatic circulation to facilitate fluid flow. It is likely that as the BMI increases, the balance between the capacity of the lymph and circulatory systems is imbalanced to a greater extent [54]. On the other hand, the results of our study may indicate that a greater amount of subcutaneous and visceral fat tissue, which is associated with central obesity, creates a physical restriction in the manual therapy. Simultaneously, it may be connected to the fact that obese patients are prone to fat necrosis, worse wound healing and various infections. Patients with obesity may have problems with muscle-pumping efficiency in the area of fat tissue. Moreover, deep lymphatic channels separated by additional subcutaneous fat may, along with abnormal body weight, lead to lack of effectiveness in compression, which then leads to limited lymphatic flow [27].

However, there are several possible explanations for the effects of MLD therapy in subjects with abnormal body weight.

First, the improvement of metabolic parameters in the subjects may be a result of the influence on the HPA axis and the autonomic nervous system. This action mechanism of MLD therapy has been confirmed by the results of studies by Shim et al. [55]. Increasing the parasympathetic activity of the autonomic nervous system plays a key role in reducing inflammation [56], reducing the risk of obesity, diabetes and chronic vascular complications [29]. Secondly, MLD therapy could increase the contractility and transport capacity of lymphatic vessels, which was the subject of several studies [30,33]. Due to the reduced ability to remove macromolecules from the interstitial space by the lymphatic system in obese people, the improvement of lymphatic drainage as a result of MLD therapy may be a desired effect in the prevention of metabolic diseases [27].

Third, 10 sessions at a physiotherapy office could have a non-specific treatment effect [57], and participation in the study itself could foster lifestyle changes that were not measured by survey questionnaires.

MLD therapy may be one of the therapies supporting the correct functioning of the lymphatic system in patients with abnormal body mass index, which may have a positive effect on selected biochemical parameters. Additionally, therapy can be a well-tolerated and relatively inexpensive method of improving the function of the lymphatic system to reduce mild chronic inflammation associated with abnormal body weight.

## 5. Study Limitations

Our research has some limitations that we would like to share. First, our study did not include patients without MLD; therefore, a placebo effect cannot be ruled out. Second, 10 sessions at a physiotherapy office could have a non-specific treatment effect, and participation in the study itself could be conducive to lifestyle changes that were not measured by survey questionnaires. Third, due to the lack of other, previous studies, tests were not used in the present study to determine sample size with a high level of confidence. Finally, our findings require confirmation in more patients with abnormal body weight.

## 6. Conclusions

Our results showed that 10 manual lymphatic drainage therapy sessions significantly decreased glycated hemoglobin, 2h-post-load glucose and high-sensitivity C-reactive protein levels in patients with an abnormal body mass index. Further studies in a larger number of patients are warranted to confirm our findings, to test in-depth their mechanism, and to investigate the clinical benefits of this alternative therapy in patients with an abnormal body mass index.

## Figures and Tables

**Table 1:** Characteristics of the subjects (*n* = 30). Data are presented as mean values and their standard deviations.

Parameter	Subjects
Age [years]	40 ± 12
SBP [mmHg]	120 ± 5
DBP [mmHg]	88 ± 8
BMI [kg/m^2^]	27 ± 5
WHR	0.92 ± 0.16
Visceral adipose tissue [LVL]	7 ± 4
FPG [mg/dL]	91 ± 15
2h-PG [mg/dL]	93 ± 20
Insulin [uIU/mL]	10 ± 8.0
C-peptide [ng/mL]	2.3 ± 1.9
HbA1c [%]	5.3 ± 0.4
hsCRP [mg/L]	2.7 ± 5
HOMA-IR	2.6 ± 2.1

Abbreviations: BMI, body mass index; DBP, diastolic blood pressure; FPG, fasting plasma glucose; HbA1c, glycated hemoglobin; HOMA-IR, homeostatic model assessment–insulin resistance; hsCRP, high-sensitivity C-reactive protein; SBP, systolic blood pressure WHR, waist–hip ratio; 2h-PG, 2h-post-load glucose.

**Table 2:** Characteristics of the anthropometric parameters of patients with normal body weight, overweight and obesity. Data are presented as mean values and their standard deviations.

Parameter	I Normal Body Weight (*n* = 14)	II Overweight (*n* = 9)	III Obesity (*n* = 7)
Age [years]	37 ± 13	40 ± 11	46 ± 10
SBP [mmHg]	120 ± 4	125 ± 3	139 ± 3
DBP [mmHg]	74 ± 6	81 ± 7	83 ± 6
Body mass index [BMI] [kg/m^2^]	22.0 ± 2.0	28.0 ± 2.0	34.0 ± 2.0
Waist–hip ratio [WHR]	0.78 ± 0.075 (normal)	1.0 ± 0.0 (abdominal obesity)	1.2 ± 0.1 (abdominal obesity)
Visceral adipose tissue [LVL]	4 ± 2	8 ± 4	12 ± 4

Abbreviations: DBP, diastolic blood pressure; SBP, systolic blood pressure.

**Table 3:** Biochemical and anthropometric parameters before and after MLD therapy in patients with normal body weight as a control group (group I). Data are presented as mean values and their standard deviations; * *p*-value for the comparison before and one month after MLD therapy.

Patients with Normal Body Weight (Group I; *n* = 14)			
Parameter	0′ (Before MLD Therapy)	1′ (One Month after MLD Therapy)	*p*-Value *
Age [years]	37 ± 13	37 ± 13	ns
BMI [kg/m^2^]	22 ± 2	22 ± 2	ns
Waist–hip ratio [WHR]	0.78 ± 0.075 (normal)	0.78 ± 0.075 (normal)	ns
Visceral adipose tissue [LVL]	4 ± 2	4 ± 2	ns
FPG [mg/dL]	85 ± 8	85 ± 10	0.960
2h-PG [ mg/dL]	77 ± 16	76 ± 15	0.864
Insulin [uIU/mL]	8.3 ± 7.8	4.9 ± 1.7	0.122
C-peptide [ng/mL]	1.9 ±1.8	1.3 ± 0.3	0.220
HbA1C [%]	5.2 ± 0.4	5.1 ± 0.4	0.613
hsCRP [mg/L]	1.0 ± 0.1	1.0 ± 0.2	0.562
HOMA-IR	2 ± 2	1.1 ± 0.4	0.112

Abbreviations: BMI, body mass index; FPG, fasting plasma glucose; HbA1c, glycated hemoglobin; HOMA-IR, homeostatic model assessment–insulin resistance; hsCRP, high-sensitivity C-reactive protein; WHR, waist–hip ratio; 2h-PG, 2h-post-load glucose; 0′, before therapy; 1′, one month after MLD therapy.

**Table 4:** Biochemical parameters before (0′) and one month after (1′) MLD therapy in a patients with overweight (group II).

Patients with Overweight (Group II)			
Parameter	0′ (Before MLD Therapy)	1′ (One Month after MLD Therapy)	*p*-Value *
Age [years]	40 ± 11	40 ± 11	ns
Body mass index BMI [kg/ m^2^]	28.0 ± 2.0	28.0 ± 2.0	ns
Waist–hip ratio [WHR]	1.0 ± 0.0 (abdominal obesity)	1.0 ± 0.0 (abdominal obesity)	ns
Visceral adipose tissue [LVL]	8 ± 4	8 ± 4	ns
FPG [mg/dL]	97 ± 9	100 ± 10	0.661
2h-PG [mg/dL]	112 ± 12	100 ± 11	0.050 *
Insulin [uIU/mL]	12.0 ± 10.9	10 ± 4.7	0.544
C-peptide [ng/mL]	3.0 ± 2.6	2.0 ± 0.7	0.390
HbA1c [%]	5.3 ± 0.3	5.3 ± 0.3	0.931
hsCRP [mg/L]	5.2 ± 8.4	2.3 ± 1.7	0.041 *
HOMA-IR	3.1 ± 2.5	2.5 ± 1.1	0.493

Data are presented as mean values and their standard deviations; * *p*-value—significant difference (*p* < 0.05) *t*-test for dependent samples. Abbreviations: BMI, body mass index; FPG, fasting plasma glucose; HbA1c, glycated hemoglobin; HOMA-IR, homeostatic model assessment–insulin resistance; hsCRP, high-sensitivity C-reactive protein; WHR, waist–hip ratio; 2h-PG, 2h-post-load glucose; 0′, before therapy; 1′, one month after MLD therapy.

**Table 5:** Biochemical parameters before (0′) and one month after (1′) MLD therapy in a patients with obesity (group III).

Patients with Obesity (Group III)			
Parameter	0′ (before MLD Therapy)	1′ (One Month after MLD Therapy)	*p*-Value *
Age [years]	46 ± 10	46 ±10	ns
Body mass index (BMI) [kg/ m^2^]	34.0 ± 2.0	34.0 ± 2.0	ns
Waist–hip ratio [WHR]	1.2 ± 0.1 (abdominal obesity)	1.2 ± 0.1 (abdominal obesity)	ns
Visceral adipose tissue [LVL]	12 ± 4	12 ± 4	ns
FPG [mg/dL]	97 ± 26	94 ± 18	0
2h-PG [mg/dL]	99 ± 14	106 ± 18	0.433
Insulin [uIU/mL]	11.0 ± 3.8	11.5 ± 4.3	0.852
C-peptide [ng/mL]	2.3 ± 0.8	2.3 ± 1.0	0.950
HbA1c [%]	5.4 ± 0.6	5.2 ± 0.6	0.013*
hsCRP [mg/L]	2.5 ± 2.9	4.0 ± 4.0	0.480
HOMA-IR	3.5 ± 2.0	3.2 ± 1.1	0.581

Data are presented as mean values and their standard deviations; * *p*-value—significant difference (*p* < 0.05) *t*-test for dependent samples. Abbreviations: Ns, not statistically significant; BMI, body mass index; FPG, fasting plasma glucose; HbA1c, glycated hemoglobin; HOMA-IR, homeostatic model assessment–insulin resistance; hsCRP, high-sensitivity C-reactive protein; WHR, waist–hip ratio; 2h-PG, 2h-post-load glucose; 0′, before therapy; 1′, one month after MLD therapy.

## Data Availability

The data presented in this study are available on request from the corresponding author: klaudia.antoniak@gumed.edu.pl.

## Supplementary Materials

The following supporting information can be downloaded at: https://www.mdpi.com/article/10.3390/biomedicines10071730/s1, Figure S1, A. Correlation between the concentration of C-peptide [ng/mL] and BMI [kg/m^2^] before and after MLD therapy, B. Correlation between the concentration of C-peptide [ng/mL] and the level of visceral adi-pose tissue before and after MLD therapy; Figure S2, A. Correlation between the level of HOMA-IR and BMI [kg/m^2^], B. Correlation between the level of HOMA-IR and visceral adipose tissue level; Figure S3, A. The level of the 2h-PG at points 0′ and 1′in group II after the use of manual lymphatic drainage therapy, B the level of the hsCRP at points 0′ and 1′; Figure S4. The level of the HbA1c at points 0′ and 1′ in group III after the use of manual lymphatic drainage therapy; Table S1: Average differences in 2h-PG levels in the group I, II and III before and after the use of MLD therapy; Table S2: Average differences in the hsCRP levels in the group I, II and III before and after the use of MLD therapy; Table S3: Average differences in the hsCRP levels in the group I, II and III before and after the use of MLD therapy.

## Abbreviations

ADA	American Diabetes Association
BMI	body mass index
CVD	cardiovascular disease
DECODE	Diabetes Epidemiology: Collaborative Analysis of Diagnostic Criteria in Europe
DBP	diastolic blood pressure
eNOS	endothelial nitric oxide synthase
FPG	fasting plasma glucose
HbA1c	glycated hemoglobin
HOMA-IR	homeostatic model assessment–insulin resistance
hsCRP	high-sensitivity C-reactive protein
IGT	impaired glucose tolerance
IL-6	interleukin-6
IL-1β	interleukin-1β
IL-18	interleukin-18
MLD	manual lymphatic drainage
OGTT	oral glucose tolerance test
PDA	Polish Diabetes Association
SBP	systolic blood pressure
TNF-α	tumor necrosis factor-α
T1D	type 1 diabetes
T2DM	type 2 diabetes
VEGF	vascular endothelial growth factor
WHR	waist–hip ratio
2h-PG	2h-post-loadglucose
