# Supplementary material for: Significant Decrease in Glycated Hemoglobin, 2h-Post-Load Glucose and High-Sensitivity C-Reactive Protein Levels in Patients with Abnormal Body Mass Index after Therapy with Manual Lymphatic Drainage
Source: Biomedicines. 2022 Jul 18;10(7):1730. doi: 10.3390/biomedicines10071730 (PMC9313311; doi:10.3390/biomedicines10071730)
Supplement: Supplementary file 1 [file biomedicines-10-01730-s001.zip › biomedicines-1759996-supplementary.pdf]

Supplementary Figure S1

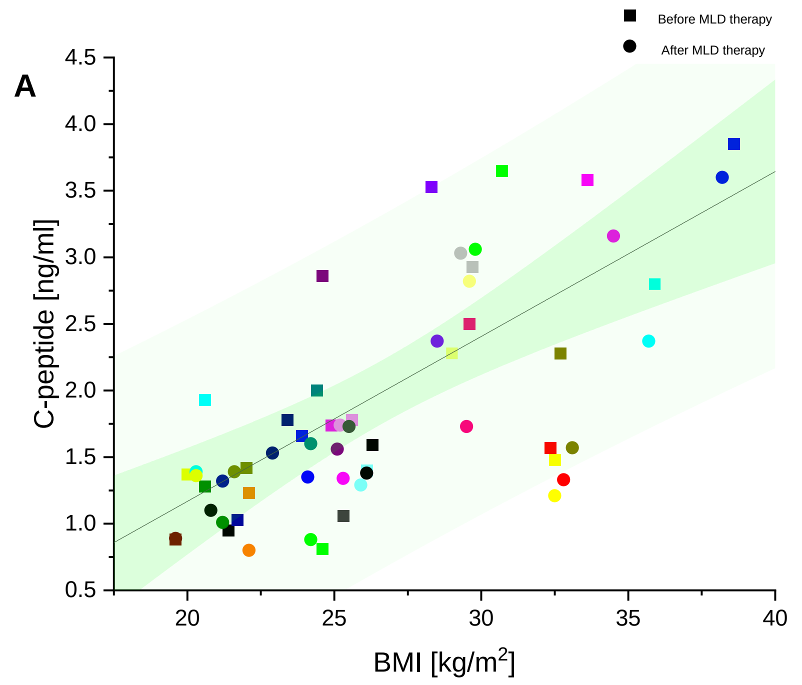

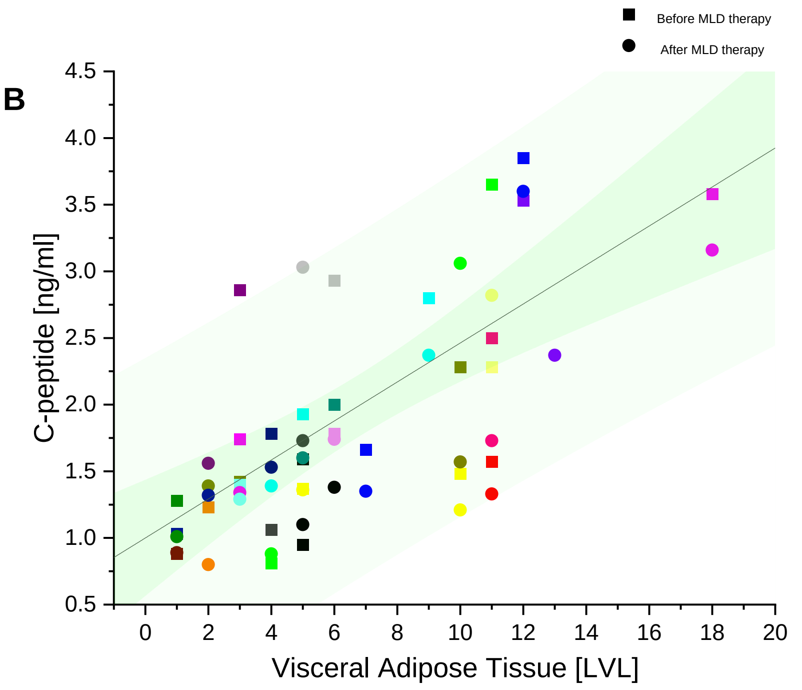

Supplementary Figure S1, A. Correlation between the concentration of C-peptide [ng/ml] and BMI [kg/m2] before and after MLD therapy (r = 0.87, p = 0.003). **Figure S1, B.** Correlation between the concentration of C-peptide [ng/mL] and the level of visceral adipose tissue before and after MLD therapy (r = 0.76, p = 0.003). A circle and a square in the same color indicate the parameters of one patient •, the concentration before MLD therapy; ▪, the concentration after MLD therapy. Abbreviations: BMI, body mass index; MLD, manual lymphatic drainage.

Supplementary Figure S2

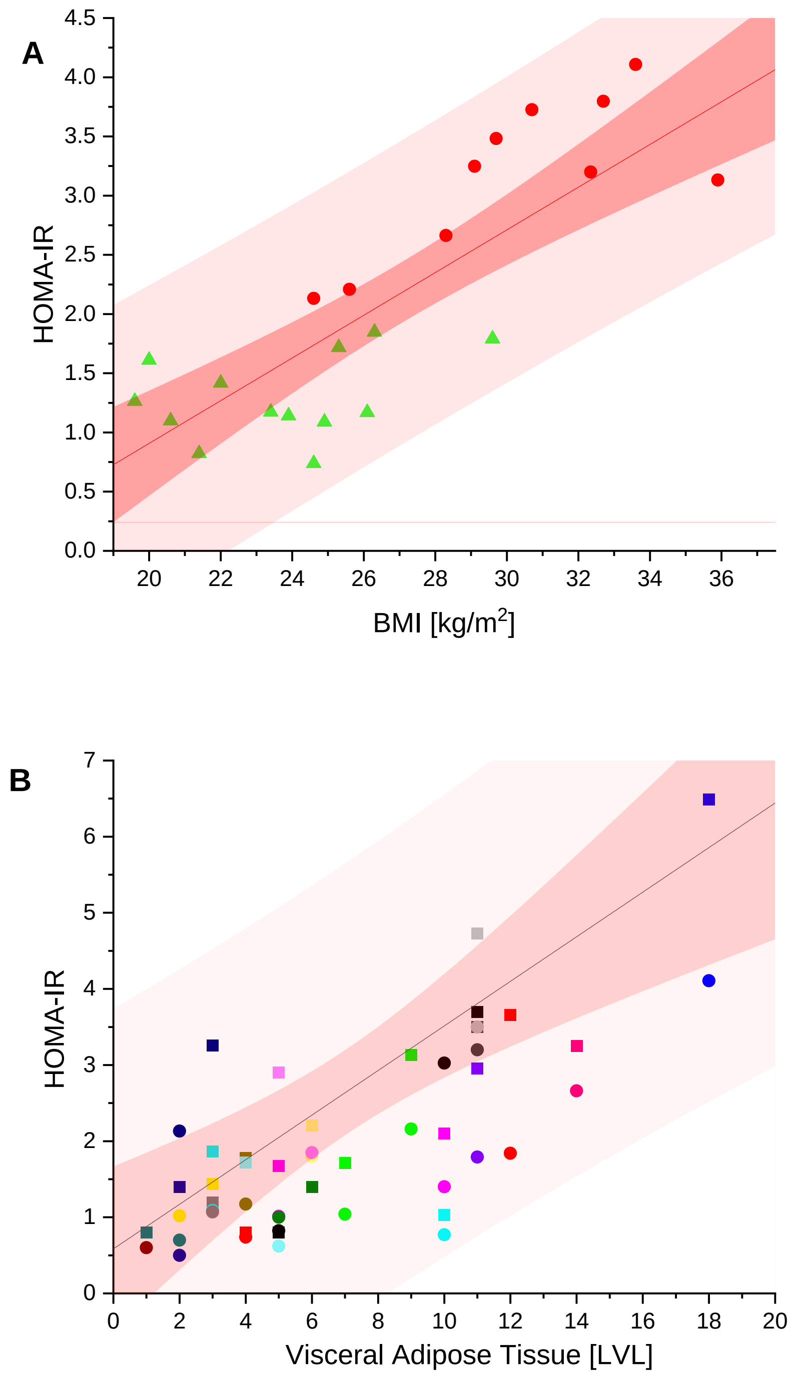

Supplementary Figure S2, A. Correlation between the level of HOMA-IR and BMI [kg/m2] (r = 0.86, *p* = 0.005). A circle and a triangle indicate the level of HOMA-IR below, equal or above to 2.0 of each patient ,▲ the level of HOMA-IR below or equal 2.0 ; • the level of HOMA-IR above 2.0
Figure S2, B. Correlation between the level of HOMA-IR and visceral adipose tissue level (r = 0.84, *p* = 0.042). A circle and a square in the same color indicate the parameters of one patient •, the concentration before MLD therapy ; ▪, the concentration after MLD therapy. Abbreviations: BMI, body mass index; MLD, manual lymphatic drainage; HOMA-IR, Homeostatic Model Assessment–Insulin Resistance.

Supplementary Figure S3

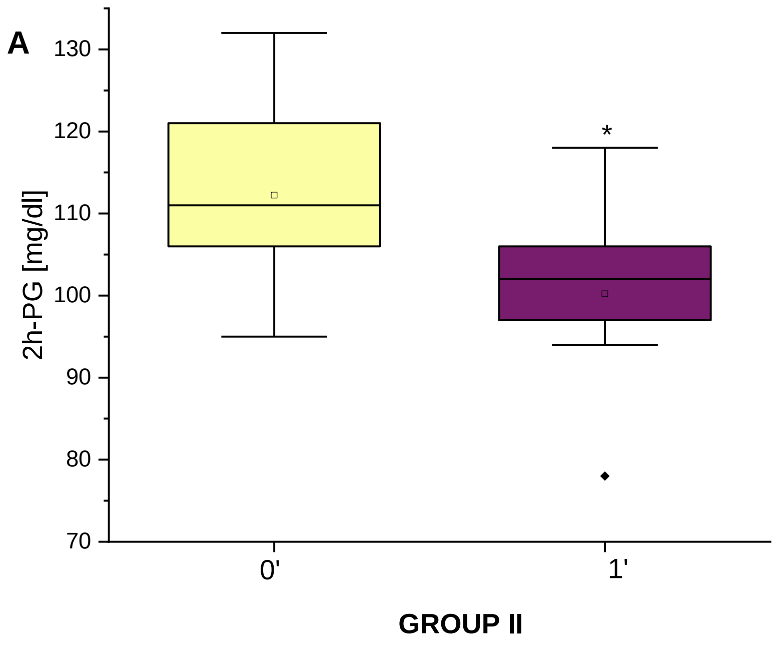

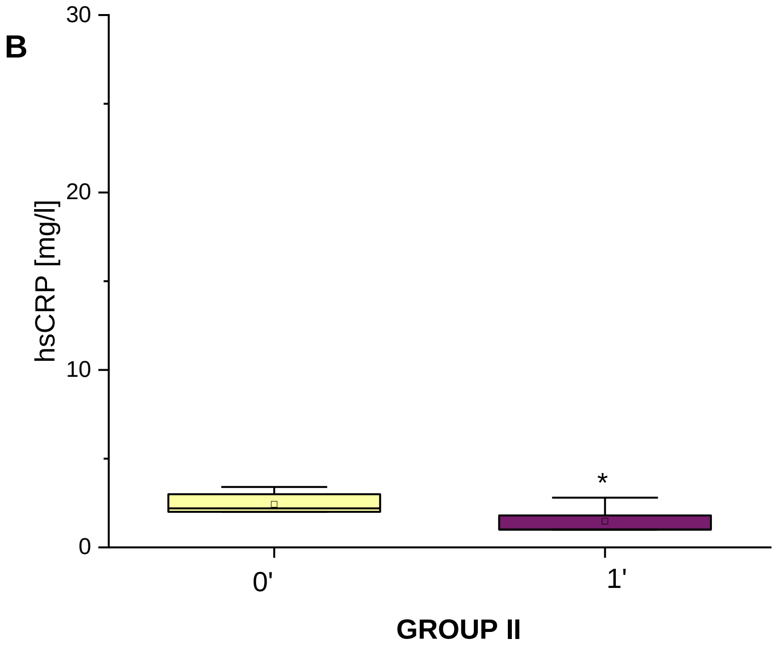

Supplementary Figure S3, A. The level of the 2h-PG at points 0′ and 1′in group II after the use of manual lymphatic drainage therapy ($p = 0.050$); ♦, patient with a different result**Figure S3, B** the level of the hsCRP at points 0′ and 1′ ($p = 0.041$), . **Abbreviations**: 0′, before therapy; 1′, one month after MLD therapy; 2h-PG, 2h-post-load glucose; hsCRP, high-sensitivity C-reactive protein; * p-value —significant difference ($p < 0.05$).

Supplementary Figure S4

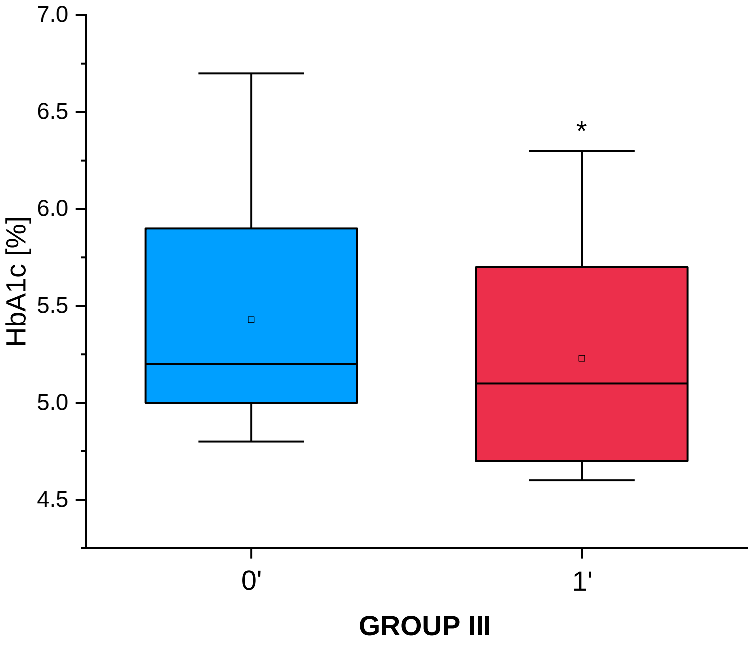

Supplementary Figure S4. The level of the HbA1c at points 0′ and 1′ in group III after the use of manual lymphatic drainage therapy ($p = 0.013$). **Abbreviations**: 0′, before therapy; 1′, one month after MLD therapy; HbA1c, glycosylated haemoglobin; * p-value —significant difference ($p < 0.05$).

Supplementary Table S1: Average differences in 2h-PG levels in the group I, II and III before and after the use of MLD therapy. The data presented are the mean; * p-value —significant difference ($p < 0.05$).

Parameter	Group I		Group II		Group III	
	**0′**	**1′**	**0′**	**1′**	**0′**	**1′**
2h-PG [mg/dL]	77	76	112	100	99	106
	**difference**		**difference**		**difference**	
	-1		-12*		+7	

Abbreviations: 0′, before therapy; 1′, one month after MLD therapy; 2h-PG, 2h-post-load glucose.

Supplementary table S2: Average differences in the hsCRP levels in the group I, II and III before and after the use of MLD therapy. The data presented are the mean; * p-value —significant difference ($p < 0.05$).

Parameter	Group I		Group II		Group III	
	**0′**	**1′**	**0′**	**1′**	**0′**	**1′**
**hsCRP [mg/L]**	1	1	5.2	2.3	2.5	4
	**difference**		**difference**		**difference**	
	0		-2.9*		+1.5	

Abbreviations: 0′, before therapy; 1′, one month after MLD therapy; hsCRP, high-sensitivity C-reactive protein.

Supplementary table S3: Average differences in the hsCRP levels in the group I, II and III before and after the use of MLD therapy. The data presented are the mean; * p-value —significant difference (p < 0.05).

Parameter	Group I		Group II		Group III	
	**0′**	**1′**	**0′**	**1′**	**0′**	**1′**
HbA1c[%]	5.3	5.3	5.2	5.1	5.4	5.2
	**difference**		**difference**		**difference**	
	0		0.1		-0.2*	

Abbreviations: 0′, before therapy; 1′, one month after MLD therapy; HbA1c, glycosylated haemoglobin.
